# Salinomycin decreases doxorubicin resistance in hepatocellular carcinoma cells by inhibiting the β-catenin/TCF complex association via FOXO3a activation

**DOI:** 10.18632/oncotarget.3585

**Published:** 2015-03-14

**Authors:** Yue Zhou, Chao Liang, Fei Xue, Wei Chen, Xiao Zhi, Xinhua Feng, Xueli Bai, Tingbo Liang

**Affiliations:** ^1^ Department of Hepatobiliary and Pancreatic Surgery, The Second Affiliated Hospital, School of Medicine, Zhejiang University, Hangzhou, PR China; ^2^ Department of Hepatobiliary and Pancreatic Surgery, People's Hospital of Zhengzhou University, School of Medicine, Zhengzhou University, Zhengzhou, PR China; ^3^ Life Sciences Institute and Innovation Center for Cell Signaling Network, Zhejiang University, Hangzhou, PR China

**Keywords:** hepatocellular carcinoma, salinomycin, doxorubicin, EMT, FOXO3a

## Abstract

Doxorubicin is a conventional and effective chemotherapy drug against hepatocellular carcinoma (HCC). However, during long-term doxorubicin monotherapy, HCC cells may eventually develop acquired-resistance to doxorubicin which results in recurrence and a poor prognosis. Salinomycin, an ionophore antibiotic, was recently reported to selectively kill human cancer stem cells (CSCs) which were response for chemoresistance. In this study, salinomycin was found to exert synergistic cytotoxicity with doxorubicin in HCC cells and be capable of inhibiting doxorubicin-induced epithelial-mesenchymal transition (EMT), an important cellular process involved in the acquired chemoresistance of tumors. Further experiments revealed that FOXO3a, a multifunctional transcription factor that can be activated by salinomycin, was vital in mediating doxorubicin-induced EMT. In addition, activated FOXO3a disturbed the interaction between β-catenin and TCF and inhibited the expression of β-catenin/TCF target genes (*ZEB1*, *c-Myc* and *CyclinD1*), which played important roles in doxorubicin-induced EMT in HCC cells. Finally, the enhanced curative efficacy of combination treatment of doxorubicin and salinomycin for HCC was confirmed in established xenograft models. In summary, the present study identifies a new doxorubicin-based chemotherapy for advanced HCC and provides a potential anti-cancer strategy targeting FOXO3a and related cell pathway molecules.

## INTRODUCTION

Hepatocellular carcinoma (HCC) is one of the most intractable cancers worldwide, and has persistently increasing rates of both incidence and mortality [1]. Treatments such as surgical resection, liver transplantation or local ablation are available for early HCC, whereas for intermediate and advanced HCC, chemotherapy may be the only approach to control tumor progression [2, 3]. Although various chemotherapies have shown some efficacy, transarterial chemoembolization (TACE) is the only treatment that has shown a consistent survival benefit in patients with intermediate HCC, and is currently considered as the standard treatment for this population in clinical guidelines [4]. Doxorubicin, an anthracycline-based agent, is one of the most widely used anti-HCC drug systematically or locally [5]; especially it is the first-line chemotherapy agent for TACE [2-4]. However, during long-term doxorubicin chemotherapy, HCC cells may eventually develop acquired chemoresistance, thereby leading to recurrence and a poor prognosis. In the treatment of multiple tumors, combining doxorubicin with other agents such as selenocystine [6], 20(S)-ginsenoside Rg3 [7], JI-34 [8] and ganetespib [9], was found to exert synergetic anticancer effects and improved doxorubicin-based chemotherapy. These reports indicated us that drug combinations may potentially be a viable strategy to improve the present situation of doxorubicin-resistance in HCC cells.

Epithelial to mesenchymal transition (EMT), a biological process during which polarized epithelial cells transform into motile mesenchymal cells, plays important roles in many processes during the progression of malignant tumors, including proliferation, invasion and migration [10]. Recent evidence indicates that acquisition of EMT features in tumor cells during chemotherapy may further promote chemoresistance [11, 12]. Moreover, related researches demonstrated that the EMT process of cancer cells was involved in the formation of chemoresistance during doxorubicin therapy in many malignant tumors, including breast cancer [13], pancreatic cancer [14] and HCC [15, 16]. Further research of the precise regulatory mechanism of doxorubicin-induced EMT, which still remains unclear, may provide a potential underlying target of overcoming doxorubicin resistance in HCC.

Salinomycin, an ionophore antibiotic, reported to selectively kill human cancer stem cells (CSCs), is considered as a promising drug to address the issue of cancer chemoresistance [17, 18]. CSCs are defined as a subpopulation of tumor cells with the capacities of self-renewal and tumor initiation, which resist conventional chemotherapy [19]. EMT is regarded as an important pathway that causes cancer cells to gain stemness and is intimately associated with tumor chemoresistance [18]. However, to date it remains inconclusive whether salinomycin is capable of regulating EMT in cancer cells. Recent research demonstrates that salinomycin downregulates the expression of EMT activators such as zinc finger E-box binding homeobox 1 (ZEB1), resulting in suppression of the EMT in mantle cell lymphoma [20]. Furthermore, the inhibitory effect of salinomycin has been reported on cell proliferation, migration and invasion in endometrial CSCs in which EMT is an important characteristic [21]. These discoveries prompted the current study to investigate the ability of salinomycin to influence the EMT process induced by doxorubicin in HCC cells and to explore a new antitumor chemotherapy.

FOXO3a, an important member of the Forkhead box O subfamily, is deemed a transcription factor at the interface of crucial cellular processes, such as metabolism, proliferation, survival and stress tolerance [22]. However, there are few reports on the function of FOXO3a during chemotherapy in HCC cells. Due to the various cellular functions mediated by FOXO3a and the lack of research in this field, the role of FOXO3a in EMT process of HCC cells was required to investigate. Hence, in this study, the ability of salinomycin to enhance the cytotoxicity of doxorubicin in HCC cells was assessed, along with the underlying mechanisms focusing on EMT and FOXO3a.

## RESULTS

### Salinomycin sensitizes HCC cells to doxorubicin *in vitro*

**Figure 1:**
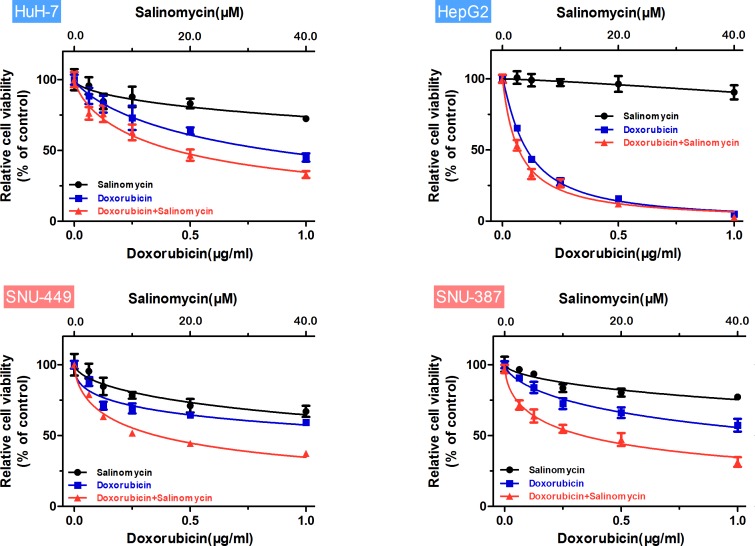
Salinomycin enhances the cytotoxicity of doxorubicin in HCC cells Relative cell viability (mean ± SD) for salinomycin (black), doxorubicin (blue) and doxorubicin plus salinomycin (red) in HuH-7, HepG2, SNU-449 and SNU-387 cells.

To investigate whether salinomycin could increase the sensitivity of HCC cells to doxorubicin therapy, CCK-8 assay was performed to measure cell viability following different treatment protocols. As shown in Fig. 1, doxorubicin plus salinomycin treatment for 48 h led to a significant decrease in cell viability compared with doxorubicin or salinomycin treatment alone in HuH-7, HepG2, SNU-449 and SNU-387 cells. To demonstrate directly whether doxorubicin and salinomycin exerted a synergetic effect with each other in HCC cells, the half maximal inhibitory concentration (IC_50_) and combination index (CI) values were calculated based on the dose-effect curves (Fig. 1). The IC_50_ values for doxorubicin at 48 h in HuH-7, HepG2, SNU-449 and SNU-387 cells were 0.86, 0.11, 1.98 and 1.47μg/ml, respectively. When used in combination with salinomycin, the IC_50_ values for doxorubicin at 48 h were significantly lower than in single use, changing into 0.43, 0.07, 0.36 and 0.34μg/ml, respectively (Table 1). In addition, the CI values for these four HCC cell lines for 48 h were 0.57, 0.68, 0.31 and 0.28, respectively, indicating synergism of doxorubicin and salinomycin (Table 1). Meanwhile, the cytotoxicity of different treatment protocols in HCC cells at more time points (12 h, 24 h and 72 h) was examined (Fig. S1). The corresponding CI values indicated additive effect of doxorubicin and salinomycin at 12 h and 72 h. And at the point of 24 h, salinomycin exerted a slight synergetic effect with doxorubicin; however, the cytotoxicity of doxorubicin did not maximize (Table S1). Hence, 48 h was selected as a reasonable time point in the following study.

**Table 1:** IC_50_ values for doxorubicin and salinomycin with HCC cells

Cell Line	IC_50_ of Sal (μM)^a^		IC_50_ of Dox (μg/ml)^b^		Combination Index
	Sal	Sal+Dox	Dox	Dox+Sal	
**HuH-7**	254.8	17.23***	0.8649	0.4306***	0.5650
**HepG2**	82.60	2.910**	0.1082	0.06864*	0.6760
**SNU-449**	113.3	14.06**	1.979	0.3596**	0.3078
**SNU-387**	261.2	13.27***	1.467	0.3446**	0.2820

a IC_50_ of salinomycin concentration in different treatments for 48 h

b IC_50_ of doxorubicin concentration in different treatments for 48 h

* p < 0.05

** p < 0.01

*** p < 0.001.

### Salinomycin inhibits EMT induced by doxorubicin in HCC cells

To investigate the influence of salinomycin on the doxorubicin-induced EMT, the morphological changes and expression of EMT markers in HCC cells treated with doxorubicin, doxorubicin plus salinomycin or salinomycin alone for 48 h were examined. It demonstrated that HuH-7 and HepG2 cells were close-connected polarized epithelial cells, doxorubicin altered them to a diffuse fibroblast-like morphology, characteristic of EMT, and salinomycin converted them back to an orderly close-connected morphology. Doxorubicin did not change the diffuse motile mesenchymal morphology of SNU-449 and SNU-387 cells, whereas salinomycin altered them to a relatively close-connected morphology (Fig. S2).

**Figure 2:**
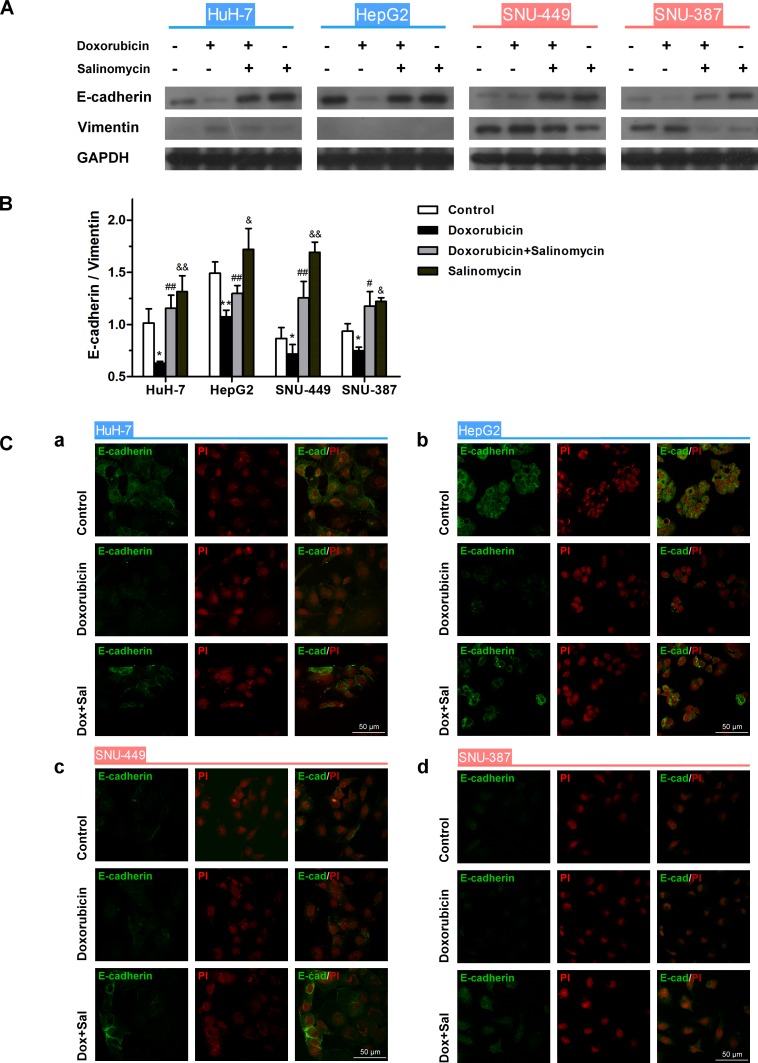
Salinomycin alters the expression of doxorubicin-induced EMT-markers in HCC cells (A) Expression of the EMT markers E-cadherin and Vimentin examined by western blotting in control HCC cells, HCC cells treated with doxorubicin (0.25μg/ml), doxorubicin (0.25μg/ml) plus salinomycin (10μM) or salinomycin (10μM) alone for 48 h. (B) Normalized ratios of E-cadherin/Vimentin calculated by analyzing the densities of western bolt bands (* p < 0.05, ** p < 0.01 for control vs. doxorubicin alone; ^#^ p < 0.05, ^##^ p < 0.01 for doxorubicin plus salinomycin vs. doxorubicin alone; ^&^ p < 0.05, ^&&^ p < 0.01 for control vs. salinomycin alone). (C) Distribution of E-cadherin detected by immunofluorescence in (a) HuH-7, (b) HepG2, (c) SNU-449 and (d) SNU-387 cells treated for 48 h with doxorubicin (0.25μg/ml) in the presence or absence of salinomycin (10μM).

Furthermore, doxorubicin significantly reduced the expression of E-cadherin and upregulated Vimentin in HCC cells compared to the untreated control, whereas salinomycin reversed doxorubicin-induced expression changes of EMT-markers (Fig. 2A and B). In addition, the expression of E-cadherin was detected by immunofluorescence, to further confirm the above-mentioned EMT in HCC cells regulated by doxorubicin and salinomycin (Fig. 2C). The results of immunofluorescent staining were consistent with the western blotting.

Moreover, cell invasion and cell migration of different HCC cells treated with doxorubicin, doxorubicin plus salinomycin or salinomycin alone for 48 h were assessed by transwell assay and wound healing assay, respectively. As shown in Fig. S3A and B, doxorubicin significantly enhanced invasion ability of HCC cells compared to the untreated control, whereas salinomycin reversed the doxorubicin-induced enhancement of cell invasion. The migration ability of HCC cells assessed by wound healing assay was consistent with the results of transwell assay (Fig. S3C and D).

### FOXO3a plays a critical role in the doxorubicin-induced EMT

**Figure 3:**
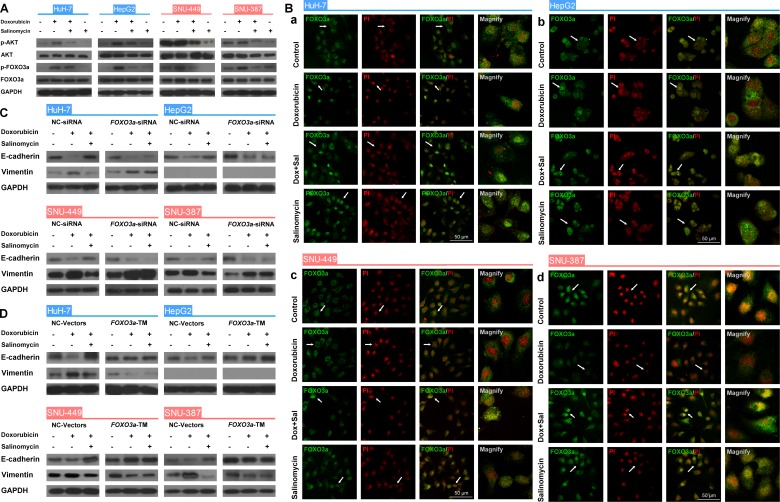
FOXO3a plays a critical role in the EMT and its activity can be regulated by doxorubicin and salinomycin (A) Western blot analysis of total-AKT, phospho-AKT (Ser473; p-AKT), total-FOXO3a and phospho-FOXO3a (Thr32; p-FOXO3a) in control HCC cells, HCC cells treated for 48 h with doxorubicin (0.25μg/ml), doxorubicin (0.25μg/ml) plus salinomycin (10μM) or salinomycin (10μM) alone. (B) The subcellular localization of FOXO3a in (a) HuH-7, (b) HepG2, (c) SNU-449 and (d) SNU-387 cells treated for 48 h with doxorubicin (0.25μg/ml), doxorubicin (0.25μg/ml) plus salinomycin (10μM) or salinomycin (10μM) alone. (C, D) Expression of the EMT markers E-cadherin and Vimentin examined by western blotting in HCC cells transfected with (C) NC-siRNA or *FOXO3a*-siRNA, (D) NC-Vector or *FOXO3a*-TM plasmid, treated with doxorubicin (0.25μg/ml) in the presence or absence of salinomycin (10μM) for 48 h.

The role of FOXO3a in the EMT process of HCC cells influenced by doxorubicin or salinomycin was then investigated. The expression of FOXO3a and phosphorated-FOXO3a was examined, as well as the subcellular localization of FOXO3a in HCC cells treated with doxorubicin, doxorubicin plus salinomycin or salinomycin alone for 48 h (Fig. 3A and B). The expression of AKT, the most important upstream regulator of the activation of FOXO3a [23, 24], was also detected. These results suggested that doxorubicin treatment significantly upregulated expression of phosphorylated FOXO3a and AKT in HCC cells, and promoted cytoplasm translocalization of FOXO3a, whereas salinomycin reversed the enhanced phosphorylation of FOXO3a and AKT and accelerated nuclear accumulation of FOXO3a (Fig. S4A and B).

Furthermore, the role of FOXO3a as the key regulator of doxorubicin-induced EMT was investigated by altering the expression of FOXO3a. *FOXO3a*-TM plasmid and *FOXO3a*-siRNA were used to upregulate and downregulate FOXO3a expression, respectively. The efficacy of *FOXO3a*-TM plasmid and *FOXO3a*-siRNA were tested in advance by measuring the relative expression levels of FOXO3a via western blotting (Fig. S5A and B). After upregulating or downregulating the active FOXO3a, the expression of EMT markers in HCC cells treated with vehicle control, doxorubicin alone or doxorubicin plus salinomycin for 48 h was examined. The results demonstrated that in HCC cells with *FOXO3a* knocked down, salinomycin failed to reverse expression changes of EMT-markers induced by doxorubicin (Fig. 3C). Meanwhile in HCC cells that overexpressed active FOXO3a, whether combined with salinomycin or not, doxorubicin could not induce significant changes in the expression of EMT-makers (Fig. 3D). In addition, CCK-8 assay was performed to investigate whether salinomycin enhanced cytotoxicity of doxorubicin in HCC cells with upregulation or downregulation of active FOXO3a. In HCC cells transfected by *FOXO3a*-siRNA, salinomycin failed to enhance the cytotoxicity of doxorubicin (Fig. S6A). In contrast, in HCC cells transfected by *FOXO3a*-TM plasmid, doxorubicin alone exerted similar cytotoxicity as when combined with salinomycin (Fig. S6B). The CI values in different HCC cells were also calculated based on the dose-effect curves in Fig. S6A and S6B. All the CI values were greater than 0.9, indicating an additive effect of doxorubicin and salinomycin (Tables 2 and 3).

**Table 2:** IC_50_ values for doxorubicin in the presence or absence of salinomycin

Cell Line	NC-siRNA			*FOXO3a*-siRNA		
	IC_50_ of Dox^a^ (μg/ml)		Combination Index	IC_50_ of Dox^b^ (μg/ml)		Combination Index
	Dox	Dox+Sal		Dox	Dox+Sal	
**HuH-7**	0.2168	0.1366***	0.6777	0.2730	0.2503**	1.042
**HepG2**	0.1151	0.07294***	0.6326	0.1335	0.1307*	0.9791
**SNU-449**	1.744	0.4690***	0.4603	1.986	1.391**	1.095
**SNU-387**	1.465	0.5311***	0.4066	2.191	1.629*	1.042

a IC_50_ of doxorubicin concentration in two treatments for 48 h for HCC cells with normal expression of FOXO3a

b IC_50_ of doxorubicin concentration in two treatments for 48 h for HCC cells with low expression of FOXO3a.

* p < 0.05

** p < 0.01

*** p < 0.001.

**Table 3:** IC_50_ values for doxorubicin treatment in the presence or absence of salinomycins

Cell Line	NC-Vectors			*FOXO3a*-TM Plasmid		
	IC_50_ of Dox^a^ (μg/ml)		Combination Index	IC_50_ of Dox^b^ (μg/ml)		Combination Index
	Dox	Dox+Sal		Dox	Dox+Sal	
**HuH-7**	0.7769	0.5072***	0.7476	0.6381	0.5065**	0.9372
**HepG2**	0.1772	0.1283**	0.7243	0.1250	0.1180*	0.9458
**SNU-449**	2.273	0.5713***	0.4173	0.4825	0.4117**	0.9962
**SNU-387**	1.459	0.5063***	0.5743	0.5811	0.4973***	1.087

a IC_50_ of doxorubicin concentration in two treatments for 48 h for HCC cells with normal expression of FOXO3a

b IC_50_ of doxorubicin concentration in two treatments for 48 h for HCC cells with high expression of FOXO3a.

* p < 0.05

** p < 0.01

*** p < 0.001.

Recent studies have demonstrated that FOXO3a competed with T-cell factor (TCF) for binding to β-catenin, an important signaling molecule in the canonical Wnt pathway, in colon carcinoma cells [25]. Whether FOXO3a regulating doxorubicin-induced EMT in HCC via changing the interaction of β-catenin/TCF4 complex, which was involved in the EMT process of various cancers [26, 27], was investigated in the following study.

### The β-catenin/TCF complex is absolutely essential to the doxorubicin-induced EMT

**Figure 4:**
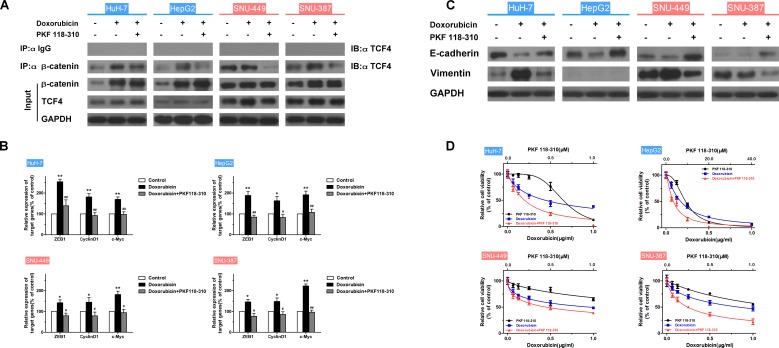
The β-catenin/TCF complex is indispensable to the doxorubicin-induced EMT (A) Endogenous β-catenin was immunoprecipitated and binding of TCF4 to β-catenin was analyzed by immunoblotting for TCF4 in HCC cells treated for 48 h with doxorubicin (0.25μg/ml) in the presence or absence of PKF 118-310 (0.5μM). (B) Normalized mRNA expression levels of the β-catenin/TCF complex target genes, *ZEB1*, *CyclinD1* and *c-Myc*, measured by RT-PCR in HCC cells treated for 48 h with doxorubicin (0.25μg/ml) in the presence or absence of PKF 118-310 (0.5μM) (* p < 0.05, ** p < 0.01 for control vs. doxorubicin alone; ^#^ p < 0.05, ^##^ p < 0.01 for doxorubicin plus PKF 118-310 vs. doxorubicin alone). (C) Expression of the EMT markers E-cadherin and Vimentin examined by western blotting in control HCC cells and HCC cells treated for 48 h with doxorubicin (0.25μg/ml) in the presence or absence of PKF 118-310 (0.5μM). (D) Relative cell viability (mean ± SD) for PKF 118-310 (black), doxorubicin (blue) and doxorubicin plus PKF 118-310 (red) in HuH-7, HepG2, SNU-449 and SNU-387 cells.

To examine the role of the β-catenin/TCF complex in doxorubicin-induced EMT, the expression of β-catenin, EMT-markers and interaction of TCF4 with β-catenin in HCC cells treated with doxorubicin in the presence or absence of PKF 118-310, a small-molecule antagonist of the β-catenin/TCF4 complex [28], was assessed. In addition, the mRNA expression levels of TCF4-dependent genes, such as *ZEB1* [29], *CyclinD1* and *c-Myc* [30, 31] were detected by RT-PCR. As shown in Figs. 4A and S7A, increased expression of β-catenin and β-catenin/TCF4 complex was observed in mesenchymal cells like SNU-449 and SNU-387, compared to epithelial cells. As expected, doxorubicin increased the β-catenin/TCF4 interaction in HCC cells, whereas PKF 118-310 disrupted the β-catenin/TCF complex. Furthermore, β-catenin/TCF complex target genes (*ZEB1*, *CyclinD1* and *c-Myc*,) which were increased by doxorubicin, were downregulated following the disruption of β-catenin/TCF4 interaction in HCC cells (Fig. 4B). The doxorubicin-induced expression changes of E-cadherin and Vimentin in HCC cells could also be reversed by PKF 118-310 (Fig. 4C). In addition, CCK-8 assay was performed to detect whether the acquired-resistance of doxorubicin in HCC cells was improved after disrupting the β-catenin/TCF complex by PKF 118-310. As shown in Fig. 4D, combined chemotherapy for 48 h led to a significant decrease in cell viability compared with doxorubicin alone. The calculated CI values of HuH-7, HepG2, SNU-449 and SNU-387, were 0.77, 0.87, 0.65 and 0.44, respectively, indicating synergism of doxorubicin and β-catenin/TCF4 complex antagonist (Table 4).

**Table 4:** IC_50_ values for doxorubicin and PKF 118-310

Cell Line	IC_50_ of PKF (μM)^a^		IC_50_ of Dox (μg/ml)^b^		Combination Index
	PKF	PKF+Dox	Dox	Dox+PKF	
**HuH-7**	0.6429	0.1964***	0.4287	0.1964**	0.7662
**HepG2**	0.2231	0.08287**	0.1677	0.08287**	0.8657
**SNU-449**	2.704	0.4632**	0.9750	0.4632**	0.6458
**SNU-387**	1.525	0.2412**	0.8539	0.2412**	0.4430

a IC_50_ PKF 118-310 concentration in two treatments for 48 h

b IC_50_ of doxorubicin concentration in two treatments for 48 h

** p < 0.01

*** p < 0.001.

### FOXO3a reduces binding of β-catenin to TCF and inhibits β-catenin/TCF target genes in HCC cells

**Figure 5:**
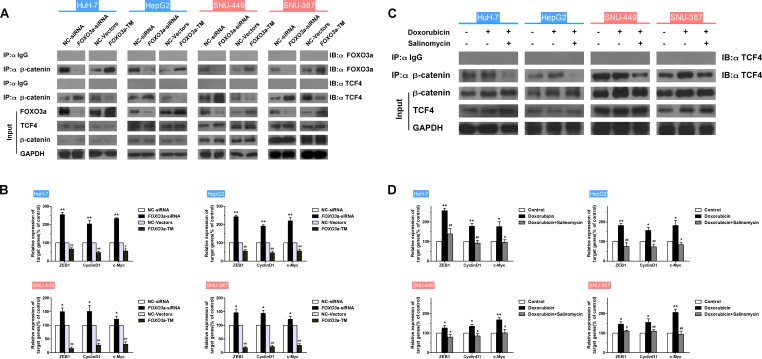
FOXO3a reduces binding of β-Catenin to TCF and inhibits expression of β-Catenin/TCF target genes in HCC cells (A) HCC cells were transfected with *FOXO3a*-siRNA or *FOXO3a*-TM plasmid. Endogenous β-catenin was immunoprecipitated, then binding of FOXO3a and TCF4 to β-catenin was analyzed by immunoblotting for FOXO3a and TCF4. (B) Normalized mRNA expression levels of the β-catenin/TCF complex target genes, *ZEB1*, *CyclinD1* and *c-Myc*, were measured by RT-PCR in HCC cells transfected with *FOXO3a*-siRNA or *FOXO3a*-TM plasmid (* p < 0.05, ** p < 0.01 for NC-siRNA vs. *FOXO3a*-siRNA; ^#^ p < 0.05, ^##^ p < 0.01 for NC-Vectors vs. *FOXO3a*-TM plasmid). (C) Endogenous β-catenin was immunoprecipitated, and binding of TCF4 to β-catenin was analyzed by immunoblotting for TCF4 in HCC cells treated for 48 h with doxorubicin (0.25μg/ml) in the presence or absence of salinomycin (10μM). (D) Normalized mRNA expression levels of the β-catenin/TCF complex target genes, *ZEB1*, *CyclinD1* and *c-Myc*, measured by RT-PCR in HCC cells treated for 48 h with doxorubicin (0.25μg/ml) in the presence or absence of salinomycin (10μM) (* p < 0.05, ** p < 0.01 for control vs. doxorubicin alone; ^#^ p < 0.05, ^##^ p < 0.01 for doxorubicin plus salinomycin vs. doxorubicin alone).

Since the critical role of the β-catenin/TCF complex in doxorubicin-induced EMT was confirmed above, the ability of FOXO3a to affect the interaction of the β-catenin/TCF complex and the β-catenin/TCF target genes was further investigated. Binding of FOXO3a and TCF4 to β-catenin in HCC cells transfected with *FOXO3a*-siRNA or *FOXO3a*-WT plasmid was assessed, and mRNA expression levels of β-catenin/TCF target genes were measured. Results showed that upregulation of FOXO3a led to increased β-catenin/FOXO3a interaction, decreased β-catenin/TCF4 interaction and reduction of mRNA expression of TCF4 target genes, meanwhile downregulation of FOXO3a led to the opposite changes (Figs. 5A, B and S7B). In addition, the binding of TCF4 to β-catenin in HCC cells treated with doxorubicin was assessed in the presence or absence of salinomycin for 48 h, and mRNA expression levels from β-catenin/TCF target genes were measured. As expected, doxorubicin caused increased β-catenin/TCF4 interaction and enhanced expression of TCF4 target genes, whereas salinomycin reversed these changes (Figs. 5C, D and S7C). Taken together these data suggest that FOXO3a and TCF4 compete for binding to β-catenin, and FOXO3a inhibits the transcription of TCF4-dependent genes which were reported relative with EMT in HCC cells.

### Salinomycin enhances the curative efficacy of doxorubicin for HCC *in vivo*

**Figure 6:**
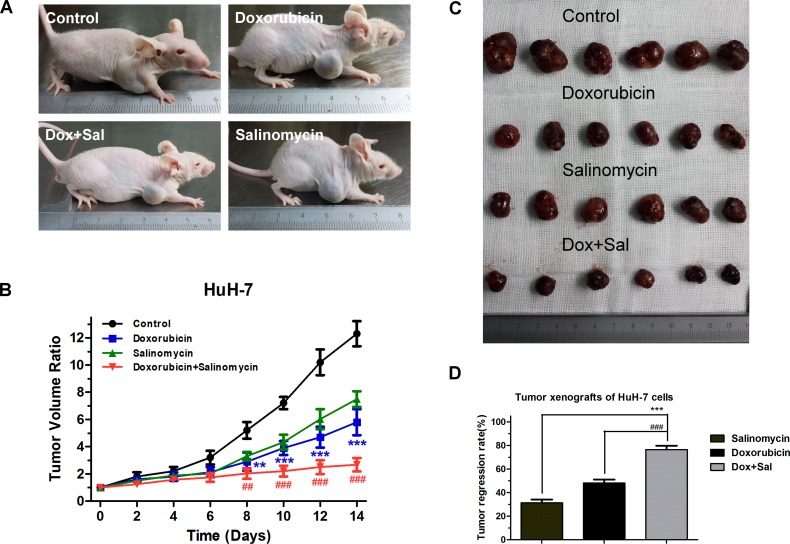
Salinomycin enhances the efficacy of doxorubicin in subcutaneous xenografts of HCC cells in nude mice (A) Representative pictures of mouse xenografts captured after 2 weeks of treatment. (B) Volume of tumor xenografts in the control group (black), groups treated with doxorubicin (blue), salinomycin (green) or doxorubicin plus salinomycin (red). Relative tumor volume ratios (% of original volume when therapy initiated) were presented as the mean ± SD, n = 6 (** p < 0.01, *** p < 0.001, for control vs. doxorubicin alone; ^##^ p < 0.01, ^###^ p < 0.001, for doxorubicin plus salinomycin vs. doxorubicin alone). (C) After 2 weeks of treatment, mice of different groups were euthanized and tumors were dissected. (D) Tumor regression rates were calculated and compared with the combination group. Results are presented as the mean ± SD, n=6 (*** p < 0.001, for doxorubicin plus salinomycin vs. salinomycin alone; ^###^ p < 0.001, for doxorubicin plus salinomycin vs. doxorubicin alone).

To investigate the *in vivo* effects of doxorubicin and salinomycin combined therapy for HCC, we established xenograft models via subcutaneous injection of HuH-7 cells into nude mice and monitored tumor growth under different treatments every other day. We found that intraperitoneal injection of doxorubicin or salinomycin alone for two weeks inhibited the growth of tumors, while combined treatment resulted in a significantly increased inhibition of tumor-growth (Fig. 6A and B). Following two weeks of chemotherapy, the mice were euthanized and the tumors were dissected and weighed. Tumor regression rates for different treatments were calculated, and salinomycin was found to significantly enhance the curative efficacy of doxorubicin for HCC *in vivo* as shown in Fig. 6C and D.

## DISCUSSION

Chemotherapy is an important component of postoperative or preoperative therapy for HCC, and may be the only approach for patients suffering from intermediate or advanced HCC. However, the curative effects of traditional monotherapeutic drugs in clinic like doxorubicin are not satisfactory. Recent reports demonstrated that certain agents such as selenocystine [6], 20(S)-ginsenoside Rg3 [7], and MK-2206 [32], exert synergetic anticancer effects with doxorubicin and may potentially improve doxorubicin-based chemotherapeutic efficacy in HCC treatment. This study demonstrated the synergism of salinomycin with doxorubicin for HCC cells and the underlying mechanism focused on EMT and FOXO3a.

Salinomycin, a traditional anticoccidial drug, has recently been shown to possess anticancer and anti-CSC effects, as well as the capacity to overcome multi-drug resistance [17-19]. Mechanisms to explain the specificity of salinomycin on CSCs and multidrug resistant cancer cells remain unclear. Gupta et al. observed the appearance of CSC like cells when EMT transformation proceeded in HMLER breast cancer cells [18]. This discovery demonstrated that EMT may be a vital pathway for salinomycin effects in CSCs. In fact, there have been few studies reported to investigate the relationship between salinomycin and EMT to date. In the current study, doxorubicin was observed to significantly reduce expression of E-cadherin and upregulate Vimentin in HCC cells, whereas salinomycin reversed doxorubicin-induced expression changes of EMT-markers, indicating salinomycin as an EMT suppressor.

As an important signaling molecule in crucial cellular processes, FOXO3a is involved in metabolism, protein homeostasis, damage repair, stress resistance and cell fate decisions [22]. It has been reported that FOXO3a activity is suppressed in drug-resistant cancer cells [33, 34], whereas acquired chemoresistance is intimately associated with EMT in multiple tumors [11, 12]. However, the precise role of FOXO3a in the EMT process of cancer cells still remains unclear. Ni et al. recently discovered that FOXO3a inactivation leads to decreased expression of snail family zinc finger 1 (SNAIL1), an E-cadherin repressor, resulting in EMT in renal clear cell carcinoma [35]. Activated FOXO3a has also been shown to reverse the EMT by activating ERα signaling in breast cancer cells [36]. Pharmacologic activation of AMPK suppresses EMT by modulating the Akt-MDM2-Foxo3 signaling axis in breast and prostate cancer cells [37]. However, the relationship between FOXO3a and EMT in HCC cells is not clear. Interestingly, FOXO3a activation was observed to be downregulated in doxorubicin-treated HCC cells in the current study, and salinomycin was found to enhance cytotoxicity of doxorubicin in HCC cells by preventing the EMT process via the activation of FOXO3a. Knockdown of *FOXO3a* resulted in salinomycin failing to reverse the EMT and overexpression of active FOXO3a caused doxorubicin to lose the capacity of inducing EMT in HCC cells. These results indicated that activation of FOXO3a by salinomycin is required to prevent the EMT process of HCC cells in doxorubicin treatment.

The transcriptional activity of FOXO3a is mainly regulated through post-translational modifications (PTMs) and nuclear-cytoplasmic shuttle, meanwhile PI3K/AKT pathway-regulated phosphorylation of FOXO3a is the most important type of PTMs to regulate FOXO3a subcellular localization [23, 24]. In this study, doxorubicin treatment was observed to induce upregulation of expression of activated AKT and led to retention of FOXO3a in the cytoplasm, whereas salinomycin reversed these changes. Although phosphorylation modification is the most important PTMs, nucleus/cytoplasm shuttling of FOXO3a is still affected by other mechanisms and further experiments are required to clarify the precise mechanism of regulation of activity of FOXO3a in HCC cells.

The Wnt/β-catenin signaling pathway is a key pathway in multiple aspects of cell processes, including cell proliferation, differentiation and morphogenesis [38]. The aberrant activation of Wnt/β-catenin signaling contributes to EMT via extremely complex mechanisms resulting in carcinogenesis and progression of multiple tumors [26, 27]. In the canonical Wnt pathway, Wnt ligands bind to the Frizzled/LRP co-receptor complex leading to the stabilization and nuclear translocation of β-catenin, a powerful transactivator of LEF/TCF transcription factors, which regulate important downstream target genes [38]. The β-catenin/TCF4 complex, a critical oncogenous complex, binds directly to the ZEB1 promoter and activates its transcription, thereby leading to EMT in colorectal cancer [29]. The results from the current study revealed that in HCC cells the β-catenin/TCF4 complex played critical roles in doxorubicin-induced EMT, and disruption of the β-catenin/TCF complex by PKF 118-310 could prevent the EMT process. As a conventional transcriptional activator of multiple genes, FOXO3a protein also co-operates with other transcription factors and modulates downstream effectors [39-41]. Diana et al. reported that FOXO3a competed with TCF for binding to β-catenin, thereby inhibiting TCF transcriptional activity and suppressing endogenous TCF target genes in colon carcinoma cells [25]. In HCC cells, β-catenin was also observed switching from TCF4 to FoxO3a, which contributed to the IFN-a2b-mediated effects on cellular proliferation and apoptosis [42]. The results of the current study demonstrate that along with the activation of FOXO3a by salinomycin treatment or consistent activation of FOXO3a by transfected with *FOXO3a*-TM in HCC cells, the association of the β-catenin/TCF4 complex was decreased and TCF4 target genes such as *ZEB1*, *c-Myc*, *CyclinD1* were inhibited, indicating that FOXO3a functions as an inhibitor of the classical Wnt signaling pathway.

In summary, this study identified a new doxorubicin-based antitumor chemotherapy which may improve curative efficacy of chemotherapy for intermediate or advanced HCC. In addition, this study provides a potential strategy that drugs targeting FOXO3a and related cell pathway molecules will benefit for anti-cancer chemotherapy. However, the mechanism of FOXO3a inhibiting the Wnt/β-catenin signaling pathway (Fig. S8) is only a small part of the complex network of cytokines and growth factors involved, therefore further research into FOXO3a is required.

## MATERIALS AND METHODS

### Cell lines and cultures

Human hepatocarcinoma cell lines (HuH-7, HepG2, SNU-449, SNU-387) were purchased from the Shanghai Institute for Biological Science (Shanghai, China). HepG2 and HuH-7 cells were cultured in Dulbecco's Minimal Essential Medium (DMEM; Gibco; Carlsbad, CA, USA) containing 10% fetal bovine serum (FBS; Gibco), 100 U/ml penicillin, and 100 mg/ml streptomycin. SNU-449 and SNU-387 cells were cultured in RPMI 1640 Medium (Gibco) supplemented with 10% FBS and 1% penicillin/streptomycin. Cells were maintained at 37°C in 5% CO_2_ and 95% air.

### Drugs and antibodies

Salinomycin, doxorubicin and PKF 118-310 were purchased from Sigma-Aldrich (St. Louis, MO, USA). Total-FOXO3a (t-FOXO3a), phospho-FOXO3a (Thr32; p-FOXO3a), total-AKT (t-AKT), phospho-AKT (Ser473; p-AKT), E-cadherin, Vimentin, β-catenin and TCF4 primary antibodies for western blotting, immunoprecipitation or immunofluorescence were obtained from Cell Signaling (Danvers, MA, USA). GAPDH primary antibodies were obtained from Kangchen Biotechnology (Sichuan, China). The HRP-conjugated secondary antibodies, goat-anti-mouse second antibody and goat-anti-rabbit second antibody, were purchased from Beijing ZhongShan Biotechnology Company (Beijing, China). Propidium iodide (PI) and anti-rabbit Alexa Fluor 488 (AF488) secondary antibody were both purchased from Invitrogen (Carlsbad, CA, USA).

### Transfection of *FOXO3a*-siRNA

HCC Cells were transfected with scrambled negative control siRNA (NC-siRNA) or *FOXO3a*-siRNA (Cell Signaling; Danvers, MA, USA) using Lipofectamine 2000 (Invitrogen; Carlsbad, CA, USA). Opti-MEM transfection medium (Gibco) was replaced with complete culture medium 6 h after transfection, and the HCC cells were incubated for the indicated times. All experiments were performed 72 h after transfection. Transfected HCC cells were plated into 96-well plates at a density of 3000 cells/well, allowed to adhere overnight and then treated with doxorubicin (0, 0.0625, 0.125, 0.25, 0.5, 1μg/ml) or salinomycin (0, 2.5, 5, 10, 20, 40μM) prior to the subsequent experiments.

### Transfection of FOXO3a overexpression plasmid

FOXO3a overexpression plasmid, *FOXO3a*-TM, in which the three AKT phosphorylation sites are mutated encoding constitutively active FOXO3a protein, was purchased from Addgene (Cambridge, MA, USA). HCC Cells were transfected with negative control vectors (NC-Vectors) or *FOXO3a*-TM plasmid using Lipofectamine-2000 for 6h and all experiments were performed 72 h after transfection. The transfection efficacy of *FOXO3a*-siRNA and *FOXO3a*-TM plasmid were assessed by examining the relative FOXO3a expression levels by western blotting.

### Cell viability assay

Cell viability was measured using Cell Counting Kit-8 (CCK-8; Dojindo; Kumamoto, Japan) following the manufacturer's instructions. HCC cells were plated into 96-well plates (3000 cells/well; 100μl media). Following overnight cultured, the media was removed and replaced with conditioned media containing different concentrations of doxorubicin (0, 0.0625, 0.125, 0.25, 0.5, 1μg/ml), salinomycin (0, 2.5, 5, 10, 20, 40μM) or PKF 118-310 (0, 0.0625, 0.125, 0.25, 0.5, 1μM). The cells were cultured for 48 h, then 10μl CCK-8 solution was added per well and the plates were incubated for 3 h. Absorbance was then measured at 450 nm using a MRX II microplate reader (Dynex, Chantilly, VA, USA). Relative cell viability was determined as a percentage of untreated control HCC cells.

### Combination analysis

Chou and Talalay introduced combination index (CI) for quantification of synergism or antagonism of two drugs in 1983. CI = (D)_1_/(D_x_)_1_+(D)_2_/(D_x_)_2_. Where CI <1, =1, and>1 indicate synergism, additive effect, and antagonism, respectively. In the denominator, (D_x_)_1_ is for D_1_ “alone” that inhibits a system x%, and (D_x_)_2_ is for D_2_ “alone” that inhibits a system x%. In the numerators, (D)_1_+(D)_2_ “in combination” also inhibit x%. The goal is to achieve the maximal effect of drugs tested on HCC cells, therefore we specified 50 for this value of “x”: CI = (IC_50_)_C1_/(IC_50_)_S or P_+(IC_50_)_C2_/(IC_50_)_D_. Where CI ≤0.9, >0.9 and <1.1, ≥1.1 indicate synergism, additive effect, and antagonism, respectively [43]. In the denominator, (IC_50_)_S or P_ is for salinomycin or PKF 118-310 “alone” that inhibits a system by 50%, and (IC_50_)_D_ is for doxorubicin “alone” that inhibits a system by 50%. In the numerators, (IC_50_)_C1_+(IC_50_)_C2_ “in combination” also inhibit by 50%. In addition, the half-maximal inhibitory concentration (IC_50_) was determined by fitting data to the equation: V% = 100/(1+10^[Drug]log IC50^), where V% is the percentage viability and [Drug] is the concentration (μg/ml or μM) of doxorubicin, salinomycin or PKF 118-310.

### Western blot assay

HCC cell lysates were washed twice in ice-cold PBS and resuspended in 100μl cell lysis buffer (Cell Signaling; Danvers, MA, USA) per treatment. The protein concentrations were quantified using the BCA Protein assay kit (Thermo Fisher Scientific Inc.; Rockford, IL, USA). Prepared protein lysates were mixed with NuPage loading buffer (Invitrogen; Carlsbad, CA, USA), denatured by boiling for 10 minutes, separated using 10% SDS-PAGE gels, and then transferred to polyvinylidene fluoride (PVDF) membranes (Millipore; Billerica, MA, USA). After blocking with sealed liquid for 2 h, membranes were incubated with primary anti-t-FOXO3a, anti-p-FOXO3a (Thr32), anti-t-AKT, anti-p-AKT (Ser473), anti-E-cadherin, anti-Vimentin, anti-GAPDH antibodies at 4°C overnight. The membranes were washed three times with tris buffered saline with 1% Tween 20 (TBS/T), incubated with the appropriate HRP-conjugated secondary antibody for 2 h at room temperature. Protein bands were developed by chemiluminescence (GE Healthcare; Piscataway, NJ, USA) and visualized using an autoradiography kit (Kodak; Rochester, NY, USA). Band densities were estimated using Image Pro Plus (Media Cybernetics, Inc.; Bethesda, MD, USA), while relative protein expression levels were normalized to GAPDH.

### Transwell assay

Invasion activity of cells *in vitro* was examined by their ability to pass through a gel matrix (Matrigel; BD; Franklin Lakes, NJ, USA). In brief, 6.5 mm diameter polycarbonate filters (8 μm pore) of the transwell chambers (Corning; NY, USA) for use in 24-well plates were coated with 65 ml of working matrigel solution (1:5 diluted with FBS-free DMEM) and were placed at 37°C for at least 0.5 h to dry. Cells were subjected to different treatments as indicated and were then seeded at a density of 2 × 10^5^ per chamber, and FBS-free DMEM was added into the upper compartments of the chambers. Meanwhile, the lower compartments of the chamber were filled with DMEM containing 10% FBS. After 48 h, non-invasive cells on the upper surface of the filters were removed completely by wiping the filter surface with cotton swabs. Viable invasive cells adhering to the lower surface of the filter were fixed using 4% polyphosphate formaldehyde (Beyotime; Shanghai, China). The invaded cells were stained using crystal violet (Beyotime; Shanghai, China). The number of stained cells in five 100× vision fields of each treatment was counted. Average cell numbers of these five fields were calculated and then normalized to control. All experiments were carried out in triplicate and independently repeated three times.

### Wound healing assay

Cells were subjected to different treatments as indicated and were then seeded in 6-well plates. After reaching 90% confluence, the cell monolayers were wounded with sterile plastic tips and were cultured in FBS-free DMEM. Cell migration was observed 24 h later by microscopy (Nikon; Tokyo, Japan), and the wound area was assessed by using Image-Pro Plus 6.0 software (IPP; Media Cybernetics; Acton, MA), normalized wound area was calculated by (wound area of 24 h / wound area of 0 h), which was then normalized to control.

### Immunofluorescence assay

HCC cells were plated into glass bottom cell culture dishes (10, 000 cells/dish), allowed to adhere overnight and then treated in conditioned media for 48 h. HCC cells were fixed with 4% formaldehyde before being permeabilized in 0.5% Triton X-100 for 15 min. Following blocking with 5% bovine serum albumin for 1h at room temperature, HCC cells were incubated with anti-E-cadherin or anti-FOXO3a primary antibody at 4°C overnight. After washing three times in ice-cold PBS for 5 minutes, the cells were incubated with anti-rabbit Alexa Fluor 488 secondary antibody at 4°C for 2 h, followed by incubation in PI for 5 min at room temperature for nuclear counterstain. Immunofluorescent imaging was performed using a confocal laser scanning microscope (Leica; Wetzlar, Germany). The subcellular localization of FOXO3a was quantified by calculating the percentage of nuclear localized FOXO3a of 100 cells from five randomly selected views in each sample.

### Co-immunoprecipitation assay

Prepared HCC cell lysates were incubated with anti-β-catenin primary antibodies overnight at 4°C. The immune complex was then precipitated at 4°C for 2 h by pre-washed Protein A-Sepharose beads (Santa Cruz Biotechnology; Dallas, Texas, USA). The proteins bound to β-catenin primary antibodies and Protein A-Sepharose beads were washed, separated using 10% SDS-PAGE Gels, transferred to PVDF membranes, and then incubated with anti-FOXO3a or anti-TCF4 primary antibody and HRP-conjugated secondary antibody. The immunoreactive bands were analyzed as western blots.

### Real-time reverse transcription-polymerase chain reaction (RT-PCR)

Total RNA was isolated from HuH-7, HepG2, SNU-449 and SNU-387 cells treated for 48 h using Trizol Reagent (Invitrogen; Carlsbad, CA, USA), and then reverse transcribed using Prime Script Reagent RT Kit (Takara Biotechnology Co.; Dalian, China) following the manufacturer's protocol. The PCR-primers for *FOXO3a*, *ZEB1*, *CyclinD1* and v-myc avian myelocytomatosis viral oncogene homolog (*c-Myc*) were designed and purchased from Takara. The sequences are as follows:

*FOXO3a*: (forward: 5-TGCGTGCCCTACTTCAAGGATAA-3; reverse: 5-ACAGGTTGTGCCGGATGGA-3)
*ZEB1*: (forward: 5-CTTGAACGTCACATGACATCACATA-3; reverse: 5-TCTTGCAGTTTGGGCATTCATA-3)
*CyclinD1*: (forward: 5-TACCGCACAACGCACTTTC-3; reverse: 5-AAGGGCTTCAATCTGTTCCTG-3),
*c-Myc*: (forward: 5-GCAGCTGCTTAGACGCTGGA-3; reverse: 5-CGCAGTAGAAATACGGCTGCAC-3).

RT-PCR was performed using ABI 7900 Prism HT (Applied Biosystems Inc.; Shanghai, China) followed by melting curve analysis. *FOXO3a*, *ZEB1*, *CyclinD1* and *c-Myc* mRNA expression was normalized to *β-actin* (forward: 5-TGGCACCCAGCACAATGAA-3; reverse: 5-CTAAGTCATAGTCCGCCTAGAAGCA-3).

### Subcutaneous mouse xenograft of HuH-7 cells

Animal research was performed in compliance with the Guide for the Care and Use of the Animal Ethics Committee of Zhejiang University (Hangzhou, PR China). Male nude mice (Shanghai Experiment Animal Centre; Shanghai, China), 3-4 weeks old and weighing 15-20 g, were used in the study, raised under pathogen-free conditions with irradiated fodder. Twenty-four nude mice were injected and randomly subdivided into four groups. Prepared HuH-7 cells (1×10^6^) resuspended in 100μl PBS were injected subcutaneously into the right axillary fossa of each mouse. Tumor length (L) and width (W) were measured with sliding caliper every other day, and tumor volumes were calculated using the formula (L×W2)/2 [44]. Drug treatment was initiated when tumor volumes reached 50-100 mm^3^. Four subgroups underwent different treatments: three experimental groups including doxorubicin (4 mg/kg), salinomycin (4 mg/kg) and doxorubicin (4 mg/kg) combined with salinomycin (4 mg/kg), as well as one vehicle-treated control group (equal volume of diluents). Drugs were delivered intraperitoneally every 2 days for 2 weeks. After 2 weeks of treatment, mice were euthanized by cervical dislocation, then tumors were dissected from each mouse and tumor weights were measured. Tumor regression rate = (1−mean tumor weight of experiment group/mean tumor weight of control group) ×100% [45].

### Statistical analyses

All the experiments data were presented as the mean and standard deviation (SD) values. Statistical analysis was performed using Prism5 (Version 5.0; GraphPad; SanDiego, CA, USA). The one-way analysis of variance (ANOVA) and Bonferroni statistical tests were used to assess the significance of different treatments; statistical significance was defined as a p-value < 0.05. Each treatment was assayed in triplicate.

## SUPPLEMENTARY MATERIAL, FIGURES AND TABLE



## References

[1] Forner A, Llovet JM, Bruix J (2012). Hepatocellular carcinoma. Lancet.

[2] Schwartz M, Roayaie S, Konstadoulakis M (2007). Strategies for the management of hepatocellular carcinoma. Nat Clin Pract Oncol.

[3] Forner A, Reig ME, de Lope CR, Bruix J (2010). Current strategy for staging and treatment:the BCLC update and future prospects. Semin Liver Dis.

[4] Forner A, Gilabert M, Bruix J, Raoul JL (2014). Treatment of intermediate-stage hepatocellular carcinoma. Nat Rev Clin Oncol.

[5] Tacar O, Sriamornsak P, Dass CR (2013). Doxorubicin: an update on anticancer molecular action, toxicity and novel drug delivery systems. J Pharm Pharmacol.

[6] Fan C, Zheng W, Fu X, Li X, Wong YS, Chen T (2014). Strategy to enhance the therapeutic effect of doxorubicin in human hepatocellular carcinoma by selenocystine, a synergistic agent that regulates the ROS-mediated signaling. Oncotarget.

[7] Kim DG, Jung KH, Lee DG, Yoon JH, Choi KS, Kwon SW, Shen HM, Morgan MJ, Hong SS, Kim YS (2014). 20(S)-Ginsenoside Rg3 is a novel inhibitor of autophagy and sensitizes hepatocellular carcinoma to doxorubicin. Oncotarget.

[8] Jaszberenyi M, Rick FG, Popovics P, Block NL, Zarandi M, Cai RZ, Vidaurre I, Szalontay L, Jayakumar AR, Schally AV (2014). Potentiation of cytotoxic chemotherapy by growth hormone-releasing hormone agonists. Proc Natl Acad Sci U S A.

[9] Lai CH, Park KS, Lee DH, Alberobello AT, Raffeld M, Pierobon M, Pin E, Petricoin Iii EF, Wang Y, Giaccone G (2014). HSP-90 inhibitor ganetespib is synergistic with doxorubicin in small cell lung cancer. Oncogene.

[10] De Craene B, Berx G (2013). Regulatory networks defining EMT during cancer initiation and progression. Nat Rev Cancer.

[11] Shao DD, Xue W, Krall EB, Bhutkar A, Piccioni F, Wang X, Schinzel AC, Sood S, Rosenbluh J, Kim JW, Zwang Y, Roberts TM, Root DE (2014). KRAS and YAP1 converge to regulate EMT and tumor survival. Cell.

[12] Huang S, Holzel M, Knijnenburg T, Schlicker A, Roepman P, McDermott U, Garnett M, Grernrum W, Sun C, Prahallad A, Groenendijk FH, Mittempergher L, Nijkamp W (2012). MED12 controls the response to multiple cancer drugs through regulation of TGF-beta receptor signaling. Cell.

[13] Zhou Y, Hu Y, Yang M, Jat P, Li K, Lombardo Y, Xiong D, Coombes RC, Raguz S, Yagüe E (2014). The miR-106b~25 cluster promotes bypass of doxorubicin-induced senescence and increase in motility and invasion by targeting the E-cadherin transcriptional activator EP300. Cell Death Differ.

[14] Rhim AD, Mirek ET, Aiello NM, Maitra A, Bailey JM, McAllister F, Reichert M, Beatty GL, Rustgi AK, Vonderheide RH, Leach SD, Stanger BZ (2012). EMT and dissemination precede pancreatic tumor formation. Cell.

[15] Hu QD, Chen W, Yan TL, Ma T, Chen CL, Liang C, Zhang Q, Xia XF, Liu H, Zhi X, Zheng XX, Bai XL, Yu XZ (2012). NSC 74859 enhances doxorubicin cytotoxicity via inhibition of epithelial-mesenchymal transition in hepatocellular carcinoma cells. Cancer Lett.

[16] Lou B, Fan J, Wang K, Chen W, Zhou X, Zhang J, Lin S, Lv F, Chen Y (2013). N1-guanyl-1,7-diaminoheptane (GC7) enhances the therapeutic efficacy of doxorubicin by inhibiting activation of eukaryotic translation initiation factor 5A2 (eIF5A2) and preventing the epithelial-mesenchymal transition in hepatocellular carcinoma cells. Exp Cell Res.

[17] Sachlos E, Risueno RM, Laronde S, Shapovalova Z, Lee JH, Russell J, Malig M, McNicol JD, Fiebig-Comyn A, Graham M, Levadoux-Martin M, Lee JB, Giacomelli AO (2012). Identification of drugs including a dopamine receptor antagonist that selectively target cancer stem cells. Cell.

[18] Gupta PB, Onder TT, Jiang G, Tao K, Kuperwasser C, Weinberg RA, Lander ES (2009). Identification of selective inhibitors of cancer stem cells by high-throughput screening. Cell.

[19] Meacham CE, Morrison SJ (2013). Tumour heterogeneity and cancer cell plasticity. Nature.

[20] Sanchez-Tillo E, Fanlo L, Siles L, Montes-Moreno S, Moros A, Chiva-Blanch G, Estruch R, Martinez A, Colomer D, Győrffy B, Roué G, Postigo A (2014). The EMT activator ZEB1 promotes tumor growth and determines differential response to chemotherapy in mantle cell lymphoma. Cell Death Differ.

[21] Kusunoki S, Kato K, Tabu K, Inagaki T, Okabe H, Kaneda H, Suga S, Terao Y, Taga T, Takeda S (2013). The inhibitory effect of salinomycin on the proliferation, migration and invasion of human endometrial cancer stem-like cells. Gynecol Oncol.

[22] Eijkelenboom A, Burgering BM (2013). FOXOs: signalling integrators for homeostasis maintenance. Nat Rev Mol Cell Biol.

[23] Trotman LC, Alimonti A, Scaglioni PP, Koutcher JA, Cordon-Cardo C, Pandolfi PP (2006). Identification of a tumour suppressor network opposing nuclear Akt function. Nature.

[24] Hu MC, Lee DF, Xia W, Golfman LS, Ou-Yang F, Yang JY, Zou Y, Bao S, Hanada N, Saso H, Kobayashi R, Hung MC (2004). IkappaB kinase promotes tumorigenesis through inhibition of forkhead FOXO3a. Cell.

[25] Hoogeboom D, Essers MA, Polderman PE, Voets E, Smits LM, Burgering BM (2008). Interaction of FOXO with beta-catenin inhibits beta-catenin/T cell factor activity. J Biol Chem.

[26] Wu ZQ, Li XY, Hu CY, Ford M, Kleer CG, Weiss SJ (2012). Canonical Wnt signaling regulates Slug activity and links epithelial-mesenchymal transition with epigenetic BreastCancer 1, Early Onset (BRCA1) repression. Proc Natl Acad Sci U S A.

[27] Zhang Q, Bai X, Chen W, Ma T, Hu Q, Liang C, Xie S, Chen C, Hu L, Xu S, Liang T (2013). Wnt/beta-catenin signaling enhances hypoxia-induced epithelial-mesenchymal transition in hepatocellular carcinoma via crosstalk with hif-1alpha signaling. Carcinogenesis.

[28] Lepourcelet M, Chen YN, France DS, Wang H, Crews P, Petersen F, Bruseo C, Wood AW, Shivdasani RA (2004). Small-molecule antagonists of the oncogenic Tcf/beta-catenin protein complex. Cancer Cell.

[29] Sánchez-Tilló E, de Barrios O (2011). Siles L, Cuatrecasas M, Castells A, Postigo A. β-catenin/TCF4 complex induces the epithelial-to-mesenchymal transition (EMT)-activator ZEB1 to regulate tumor invasiveness. Proc Natl Acad Sci U S A.

[30] Kim BM, Mao J, Taketo MM, Shivdasani RA (2007). Phases of canonical Wnt signaling during the development of mouse intestinal epithelium. Gastroenterology.

[31] Trautmann M, Sievers E, Aretz S, Kindler D, Michels S, Friedrichs N, Renner M4, Kirfel J, Steiner S, Huss S, Koch A, Penzel R, Larsson O (2014). SS18-SSX fusion protein-induced Wnt/beta-catenin signaling is a therapeutic target in synovial sarcoma. Oncogene.

[32] Simioni C, Martelli AM, Cani A, Cetin-Atalay R, McCubrey JA, Capitani S, Neri LM (2013). The AKT inhibitor MK-2206 is cytotoxic in hepatocarcinoma cells displaying hyperphosphorylated AKT-1 and synergizes with conventional chemotherapy. Oncotarget.

[33] Fernandez de Mattos S, Villalonga P, Clardy J, Lam EW (2008). FOXO3a mediates the cytotoxic effects of cisplatin in colon cancer cells. Mol Cancer Ther.

[34] Ausserlechner MJ, Salvador C, Deutschmann A, Bodner M, Viola G, Bortolozzi R, Basso G, Hagenbuchner J, Obexer P (2013). Therapy-resistant acute lymphoblastic leukemia (ALL) cells inactivate FOXO3 to escape apoptosis induction by TRAIL and Noxa. Oncotarget.

[35] Ni D, Ma X, Li HZ, Gao Y, Li XT, Zhang Y, Ai Q, Zhang P, Song EL, Huang QB, Fan Y, Zhang X (2014). Downregulation of FOXO3a promotes tumor metastasis and is associated with metastasis-free survival of patients with clear cell renal cell carcinoma. Clin Cancer Res.

[36] Belguise K, Guo S, Sonenshein GE (2007). Activation of FOXO3a by the green tea polyphenol epigallocatechin-3-gallate induces estrogen receptor alpha expression reversing invasive phenotype of breast cancer cells. Cancer Res.

[37] Chou CC, Lee KH, Lai IL, Wang D, Mo X, Kulp SK, Shapiro CL, Chen CS (2014). AMPK Reverses the Mesenchymal Phenotype of Cancer Cells by Targeting the Akt-MDM2-Foxo3a Signaling Axis. Cancer Res.

[38] Clevers H, Nusse R (2012). Wnt/beta-catenin signaling and disease. Cell.

[39] Essers MA, de Vries-Smits LM, Barker N, Polderman PE, Burgering BM, Korswagen HC (2005). Functional interaction between beta-catenin and FOXO in oxidative stress signaling. Science.

[40] Cho EC, Kuo ML, Liu X, Yang L, Hsieh YC, Wang J, Cheng Y, Yen Y (2014). Tumor suppressor FOXO3 regulates ribonucleotide reductase subunit RRM2B and impacts on survival of cancer patients. Oncotarget.

[41] Liang C, Chen W, Zhi X, Ma T, Xia X, Liu H, Zhang Q, Hu Q, Zhang Y, Bai X, Liang T (2013). Serotonin promotes the proliferation of serum-deprived hepatocellular carcinoma cells via upregulation of FOXO3a. Mol Cancer.

[42] Ceballos MP, Parody JP, Quiroga AD, Casella ML, Francés DE, Larocca MC, Carnovale CE, Alvarez Mde L, Carrillo MC (2014). FoxO3a Nuclear Localization and Its Association with beta-Catenin and Smads in IFN-alpha-Treated Hepatocellular Carcinoma Cell Lines. J Interferon Cytokine Res.

[43] Chou TC (2006). Theoretical basis, experimental design, and computerized simulation of synergism and antagonism in drug combination studies. Pharmacol Rev.

[44] Naito S, von Eschenbach AC, Giavazzi R, Fidler IJ (1986). Growth and metastasis of tumor cells isolated from a human renal cell carcinoma implanted into different organs of nude mice. Cancer Res.

[45] Povlsen CO, Jacobsen GK (1975). Chemotherapy of a human malignant melanoma transplanted in the nude mouse. Cancer Res.

