# Supplementary material for: Salinomycin decreases doxorubicin resistance in hepatocellular carcinoma cells by inhibiting the β-catenin/TCF complex association via FOXO3a activation
Source: Oncotarget. 2015 Mar 14;6(12):10350–65. doi: 10.18632/oncotarget.3585 (PMC4496360; doi:10.18632/oncotarget.3585)
Supplement: Supplementary file 1 [file oncotarget-06-10350-s001.pdf]

Salinomycin decreases doxorubicin resistance in hepatocellular carcinoma cells by inhibiting the β-catenin/TCF complex association via FOXO3a activation

Supplementary Material

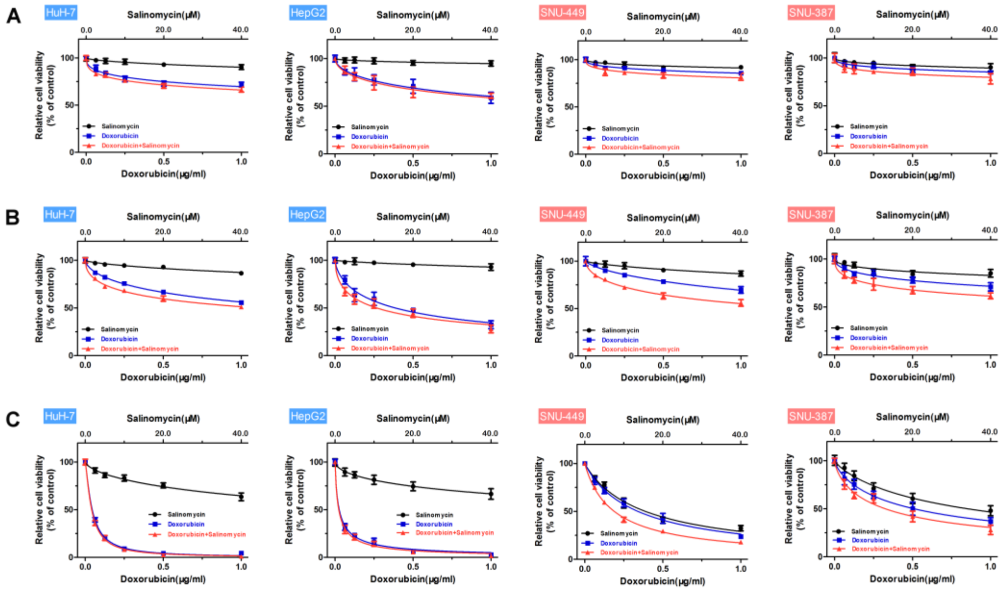

Supplementary Figure S1: Drug combination studies of doxorubicin and salinomycin at different time points in HCC cells. Relative cell viability (mean ± SD) for salinomycin (black), doxorubicin (blue) and doxorubicin plus salinomycin (red) in HuH-7, HepG2, SNU-449 and SNU-387 cell lines at 12 h(A), 24 h(B) and 72 h(C).

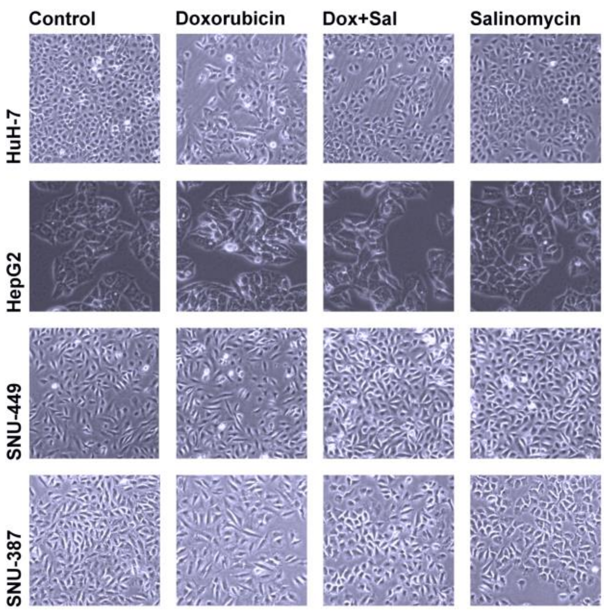

Supplementary Figure S2: Morphological changes of different HCC cells treated with doxorubicin or salinomycin. Morphological changes were observed and captured by optical microscopy in HuH-7, SNU-449, SNU-387 (×100 magnification), and HepG2 (×400 magnification) cells treated for 48 h with doxorubicin (0.25μg/ml), doxorubicin (0.25μg/ml) plus salinomycin (10μM) or salinomycin (10μM) alone.

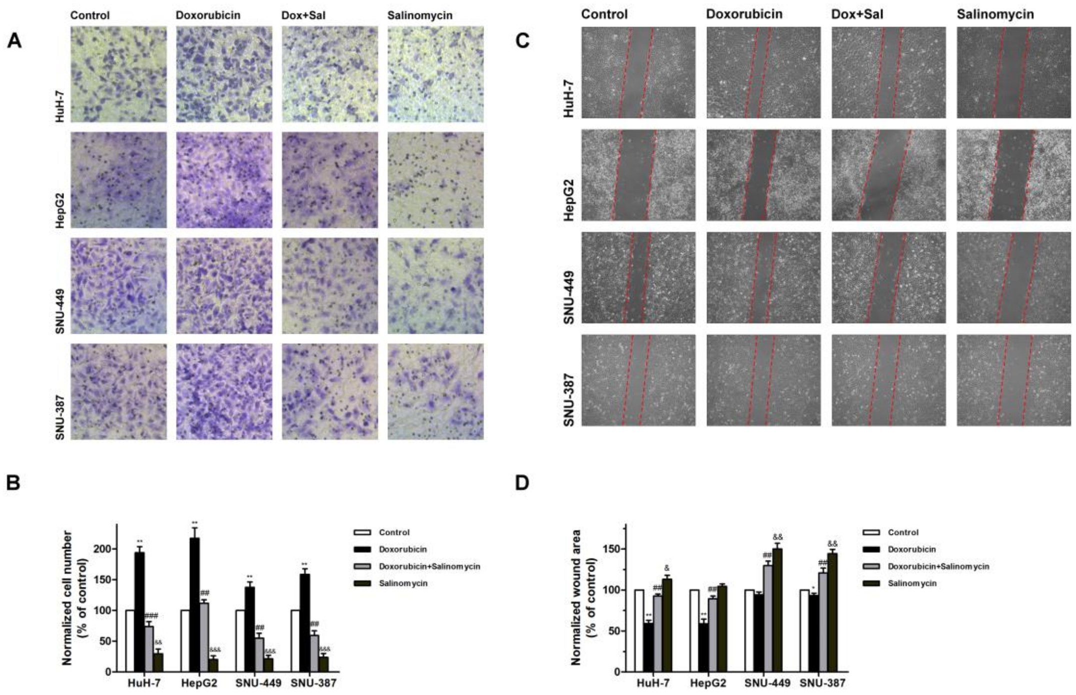

Supplementary Figure S3: Invasion and migration ability changes of different HCC cells treated with doxorubicin or salinomycin. (A) Cell invasion assessed by transwell assays in control HCC cells, HCC cells treated with doxorubicin (0.25μg/ml), doxorubicin (0.25μg/ml) plus salinomycin (10μM) or salinomycin (10μM) alone for 48 h (×100 magnification). (B) The number of stained cells in five 100× vision fields of each treatment was counted. Average cell numbers of these five fields were calculated and then normalized to control (** $p < 0.01$, for control vs. doxorubicin alone; $^{\#\#}p < 0.01$, $^{\#\#\#}p < 0.001$, for doxorubicin plus salinomycin vs. doxorubicin alone; $^{\&\&}p < 0.01$, $^{\&\&\&}p < 0.001$, for control vs. salinomycin alone). (C) Cell migration assessed by Wound healing assay in control HCC cells, HCC cells treated with doxorubicin (0.25μg/ml), doxorubicin (0.25μg/ml) plus salinomycin (10μM) or salinomycin (10μM) alone for 48 h (×100 magnification). (D) The wound

area was assessed by using Image-Pro Plus 6.0 software, normalized wound area was calculated by (wound area at 24 h / wound area at 0 h), which was then normalized to control (* $p < 0.05$, ** $p < 0.01$, for control vs. doxorubicin alone; $^{\#\#}$ $p < 0.01$, for doxorubicin plus salinomycin vs. doxorubicin alone; $^{\&}$ $p < 0.05$, $^{\&\&}$ $p < 0.01$, for control vs. salinomycin alone).

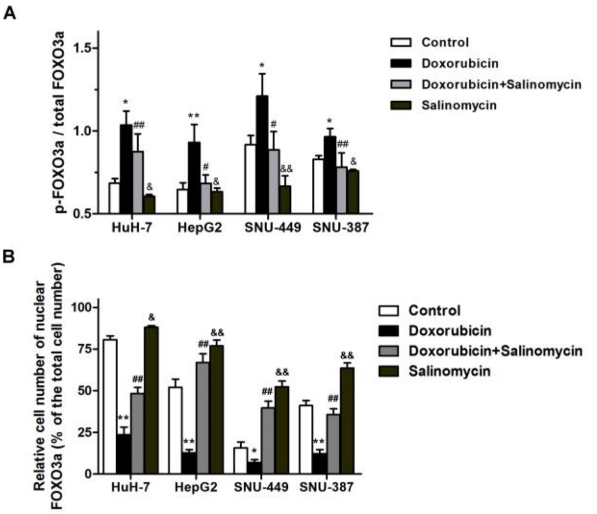

Supplementary Figure S4: The activity of FOXO3a regulated by doxorubicin or salinomycin. (A) The normalized ratios of p-FOXO3a/total-FOXO3a were calculated by analyzing the densities of western blot bands (* $p < 0.05$, ** $p < 0.01$ for control vs. doxorubicin; $^{\#}$ $p < 0.05$, $^{\#\#}$ $p < 0.01$ for doxorubicin plus salinomycin vs. doxorubicin alone; $^{\&}$ $p < 0.05$, $^{\&\&}$ $p < 0.01$ for control vs. salinomycin alone). (B) Quantification of immunofluorescence staining patterns for FOXO3a (* $p < 0.05$, ** $p < 0.01$ for control vs. doxorubicin; $^{\#\#}$ $p < 0.01$ for doxorubicin plus salinomycin vs. doxorubicin alone; $^{\&}$ $p < 0.05$, $^{\&\&}$ $p < 0.01$ for control vs. salinomycin alone).

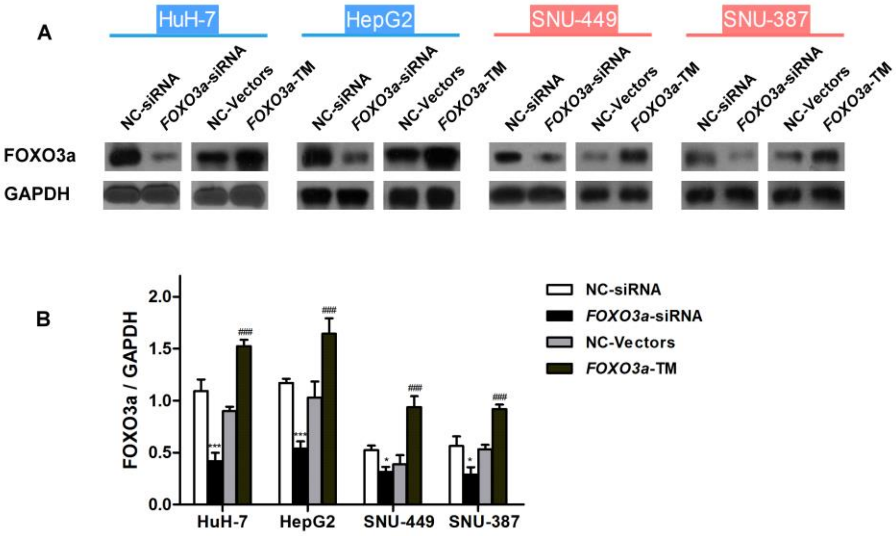

Supplementary Figure S5: Efficacy of *FOXO3a*-siRNA and *FOXO3a*-TM plasmid. (A) Relative expression levels of FOXO3a measured by western blotting in HCC cells transfected by *FOXO3a*-siRNA or *FOXO3a*-TM plasmid. (B) The ratios of FOXO3a/GAPDH were calculated by analyzing the densities of western blot bands (* $p < 0.05$, *** $p < 0.001$ for NC-siRNA vs. *FOXO3a*-siRNA; $^{\#\#\#}$ $p < 0.001$ for NC-Vectors vs. *FOXO3a*-TM plasmid).

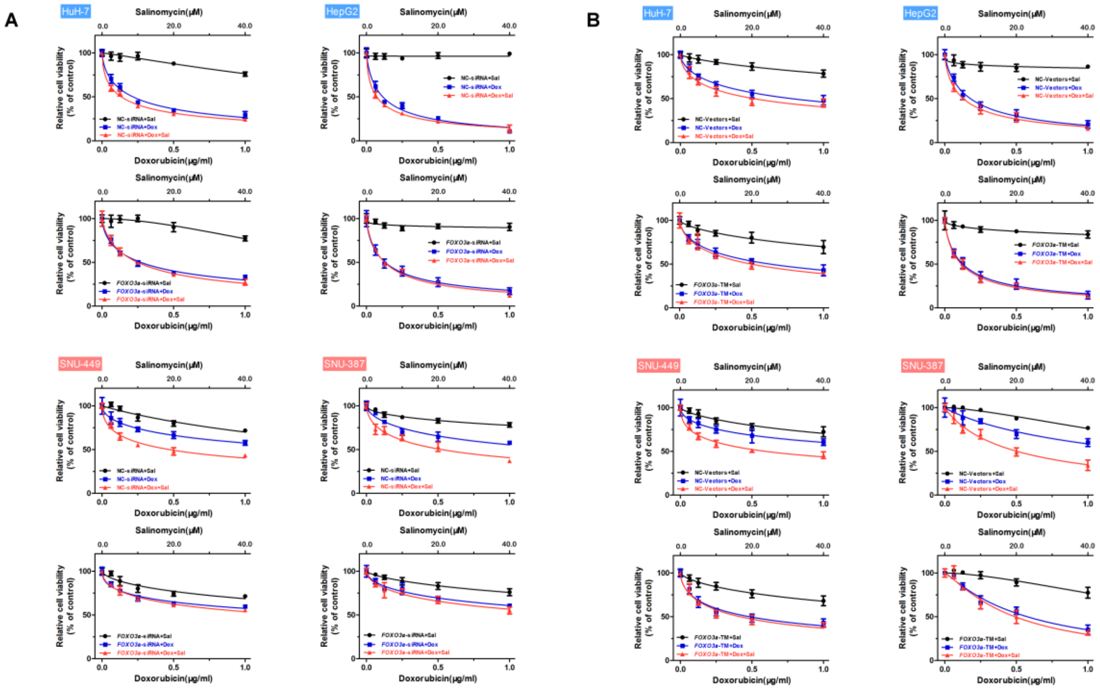

Supplementary Figure S6: The cytotoxicity of doxorubicin or salinomycin in HCC cells with up-regulation or downregulation of active FOXO3a. Relative cell viability (mean ± SD) for salinomycin (black), doxorubicin (blue) and doxorubicin plus salinomycin (red) in HuH-7, HepG2, SNU-449 and SNU-387 cells transfected with (A) NC-siRNA or *FOXO3a*-siRNA, (B) NC-Vectors or *FOXO3a*-TM plasmid.

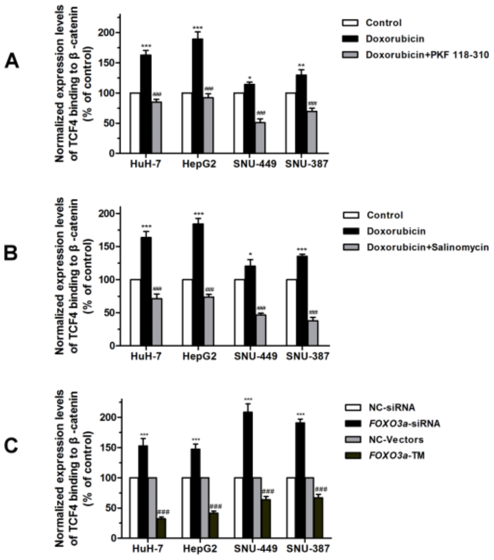

Supplementary Figure S7: Normalized expression levels of TCF4 binding to β-catenin. Normalized expression levels of TCF4 binding to β-catenin were calculated by analyzing the densities of western blot bands in HuH-7, HepG2, SNU-449 and SNU-387 cells (A) treated for 48 h with doxorubicin (0.25μg/ml) in the presence or absence of PKF 118-310 (0.5μM) (* $p < 0.05$, ** $p < 0.01$, *** $p < 0.001$ for control vs. doxorubicin alone; ### $p < 0.001$ for doxorubicin plus PKF 118-310 vs. doxorubicin alone); (B) treated for 48 h with doxorubicin (0.25μg/ml) in the presence or absence of salinomycin (10μM) (* $p < 0.05$, *** $p < 0.001$ for control vs. doxorubicin alone; ### $p < 0.001$ for doxorubicin plus salinomycin vs. doxorubicin alone); (C) transfected with NC-siRNA, *FOXO3a*-siRNA, NC-Vectors or *FOXO3a*-TM plasmid (*** $p < 0.001$ for NC-siRNA vs. *FOXO3a*-siRNA; ### $p < 0.001$ for NC-Vectors vs. *FOXO3a*-TM plasmid).

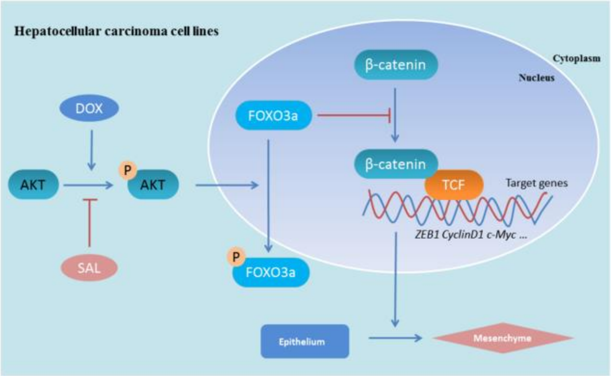

Supplementary Figure S8: The schematic diagram of mechanism of FOXO3a inhibiting Wnt/β-catenin signaling pathway in HCC cells. Salinomycin-activated FOXO3a affected the Wnt/β-catenin signaling pathway via inhibition of the β-catenin/TCF complex association and reducing the transcription of TCF target genes, thereby suppressing the doxorubicin-induced EMT in HCC cells.

Table S1: Combination Index of doxorubicin and salinomycin for HCC cells.

Cell Line	Combination Index		
	**12h** [a]	**24h** [b]	**72h** [c]
**HuH-7**	0.9162	0.8087	0.9447
**HepG2**	0.9202	0.6986	0.9241
**SNU-449**	0.9312	0.6353	1.038
**SNU-387**	1.078	0.5010	1.018

[a] Combination Index of doxorubicin and salinomycin for HCC cells at 12h;

[b] Combination Index of doxorubicin and salinomycin for HCC cells at 24h;

[c] Combination Index of doxorubicin and salinomycin for HCC cells at 72h.
